# In Silico Subtractive Proteomics Approach for Identification of Potential Drug Targets in *Staphylococcus saprophyticus*

**DOI:** 10.3390/ijerph17103644

**Published:** 2020-05-22

**Authors:** Farah Shahid, Usman Ali Ashfaq, Sania Saeed, Samman Munir, Ahmad Almatroudi, Mohsin Khurshid

**Affiliations:** 1 Department of Bioinformatics and Biotechnology, Government College University, Faisalabad, Punjab 38000, Pakistan; farah.shahid.209@gmail.com (F.S.); saniasaeed1993@yahoo.com (S.S.); sammanmunir01@gmail.com (S.M.); 2 Department of Medical Laboratories, College of Applied Medical Sciences, Qassim University, Buraydah 52571, Saudi Arabia; aamtrody@qu.edu.sa; 3 Department of Microbiology, Government College University, Faisalabad, Punjab 38000, Pakistan; mohsinkhurshid@gcuf.edu.pk

**Keywords:** subtractive proteomics, drug targets identification, bioinformatics, *Staphylococcus saprophyticus*

## Abstract

*Staphylococcus saprophyticus* is a uropathogenic bacteria responsible for acute urinary tract infections (UTIs) mainly in young female patients. Patients suffering from urinary catheterization, pregnant patients, the elderly as well as those with nosocomial UTIs are at greater risk of the colonizing *S. saprophyticus* infection. The causative factors include benign prostatic hyperplasia, indwelling catheter, neurogenic bladder, pregnancy, and history of frequent UTIs. Recent findings have exhibited that *S. saprophyticus* is resistant to several antimicrobial agents. Moreover, there is a global concern regarding the increasing level of antimicrobial resistance, which leads to treatment failure and reduced effectiveness of broad-spectrum antimicrobials. Therefore, a novel approach is being utilized to combat resistant microbes since the past few years. Subtractive proteome analysis has been performed with the entire proteome of *S. saprophyticus* strain American Type Culture Collection (ATCC) 15305 using several bioinformatics servers and software. The proteins that were non-homologous to humans and bacteria were identified for metabolic pathway analysis. Only four cytoplasmic proteins were found possessing the potential of novel drug target candidates. The development of innovative therapeutic agents by targeting the inhibition of any essential proteins may disrupt the metabolic pathways specific to the pathogen, thus causing destruction as well as eradication of the pathogen from a particular host. The identified targets can facilitate in designing novel and potent drugs against *S. saprophyticus* strain ATCC 15305.

## 1. Introduction

Urinary tract infections (UTIs) are the most common bacterial infection and a lot of women suffer from this infection at least once in a lifetime. In the USA, urinary disorders are one of the frequent complaints in people receiving medical attention. *Staphylococcus saprophyticus* has emerged as the second major cause of community-acquired UTIs, after *Escherichia coli*. Until the 1960s, coagulase-negative staphylococci were considered urinary pollutants. In 1962, the isolation of coagulase-negative staphylococci was reported by Torres Pereira. It carries 51 urine antigens of women with acute urinary tract infections [1]. Many studies backed this idea in the following years [2]. The organism ended up being part of subgroup 3 of *Micrococcus*. Later it was reclassified as *S. saprophyticus*. Another important feature is urease production and the association of ureteral and renal stones with *S. saprophyticus* infection [3,4]. Among females of the age group 16–25, *S. saprophyticus* causes approximately 42% of infections, whereas in 40% of sexually active young females, *S. saprophyticus* occurs as part of the normal urogenital tract [5,6]. More rarely, it also causes various other complications such as urethritis, prostatitis, pyelonephritis, and epididymitis [7]. *S. saprophyticus* is also a cause of urinary tract infections in men of all ages and found in juveniles, homosexual men, and aged men having urinary catheterization [8,9]. It is rarely found in hospitalized men and causes epididymitis, urethritis, and kidney stones in men [10]. *S. saprophyticus* is usually obtained from the gastrointestinal (GI) tract of humans, which can be a source for the inoculation of UTIs. Since limited data are available regarding the epidemiology of UTIs caused by *S. saprophyticus*, the main source and reservoir of this bacterial species for human complications have not been fully described. Thereby, it is currently uncertain whether or not species-specific clones or strains are associated with UTIs [11]. 

The occurrence of antibiotic resistance in bacterial uropathogens has become an international issue. The emergence of resistance in *S. saprophyticus* against the commonly prescribed antibiotics for the empiric treatment of cystitis such as Fosfomycin and Cefixime complicates the management of patients suffering from this bacterium [12].

In the modern postgenomic period, the probabilities of selecting suitable targets through computational methods and integration of “omics” data, such as proteomics, metabolomics, and genomics have been expanding continuously. The in silico approaches such as subtractive and comparative proteomics are now being used extensively for the identification as well as the prediction of drug targets for several pathogenic bacteria [13]. Compared to traditional methods, these techniques are efficient, time saving as well as cost effective in drug designing processes [14]. In the last few years, the species-specific vaccine candidates and potential drug targets have been designed for several pathogenic bacteria using the approach of subtractive proteomics [15,16].

In the present study, the proteome of *S. saprophyticus* strain ATCC 15305 was studied to employ various approaches of subtractive proteomics. Several computational software were utilized to recognize the essential proteins that are necessary for the survival of bacteria. The analysis of both the metabolic pathways and host non-homology was performed to prevent the cross-reactivity of potential drugs with bacterial and host proteins, along with the involvement of bacterial proteins in various metabolic processes of the host, respectively. The investigation was further expanded to identify the cytoplasmic proteins exhibiting uniqueness as potential drug targets. Therefore, this study will help in developing potent drug targets against *S. saprophyticus* strain ATCC 15305. 

## 2. Methodology

The entire proteome of *S. saprophyticus* strain ATCC 15305 was studied by using the approach of subtractive proteomics for identifying potential drug targets. 

### 2.1. Proteome Retrieval

The complete proteome of *S. saprophyticus* strain (ATCC 15305) was downloaded from UniProt in FASTA format. UniProt (Universal Protein Resource) is an important database that provides a reliable, accurate, and freely available central resource on protein sequences and functional annotation. The proteome can be obtained from the Proteome section of the UniProt website [17].

### 2.2. Removal of Paralog Sequences

Paralogs or redundant sequences from the *S. saprophyticus* proteome were removed by using CD-HIT at the threshold of 60%. CD-HIT is an extensively used software for clustering of biological sequences, which decreases the redundancy of protein sequences and improves the performance of other sequence analyses [18]. 

### 2.3. Retrieval of Essential Proteins

Essential proteins are considered fundamental for cell survival. These proteins were retrieved by using an online software Geptop 2.0 server. The essentiality cutoff score was set at 0.24. Geptop is a web server that provides a forum for identifying essential genes for bacterial organisms, comparing query protein orthology and phylogeny with the experimentally developed essential gene datasets (from the Database of Essential Genes DEG). Geptop can be utilized for any species of bacteria that has a sequenced genome [19]. 

### 2.4. Essential Non-Homologous Protein Identification

For the detection of the non-homologous protein sequences of *S. saprophyticus*, essential proteins were submitted against the human proteome with default values of the parameter to BlastP. The proteins exhibiting an identity of ≤30% and query coverage of >70 were labeled as non-homologous.

### 2.5. Unique Pathway Identification

Non-homologous essential proteins were computed for comparative analysis of metabolic pathways. This analysis is conducted to identify drug targets based on pathway enzymes that are both common and essential to bacteria [20]. The metabolic pathways of *S. saprophyticus* were identified by comparing the pathways of both the *S. saprophyticus* and *Homo sapiens* through the Kyoto Encyclopedia of Genes and Genomes (KEGG) database [21]. Those metabolic pathways were selected that are unique to only *S. saprophyticus* (strain ATCC 15305) and are not found in humans. Thus, the proteins having unique metabolic pathways (UMPs) were selected for further assessment.

### 2.6. Subcellular Localization Prediction

Subcellular localization prediction of proteins is significant for genome annotation and genome analysis in bacterial pathogens, as these proteins possess the potential of primary drug or vaccine targets [22]. The subcellular localization of proteins that are associated with unique metabolic pathways of *S. saprophyticus* was predicted through PSORTb and BUSCA [23,24].

### 2.7. Druggability Analysis

The druggability of potential proteins was analyzed in the DrugBank database for all drug targets. BlastP of selected proteins was performed with predefined parameters against a list of compounds found within the database of DrugBank. This is a unique tool in chemoinformatics and bioinformatics that integrates quantitative data on drugs with extensive knowledge about drug targets. This database includes 4323 non-redundant protein sequences consisting of the drug target/enzyme/transporter carrier associated with 6712 drug entries consisting of 131 FDA-approved biotech (protein/peptide) drugs (FDA: Food and drug administration), 1448 FDA-approved small-molecule drugs, 5080 experimental drugs, and 85 nutraceuticals [25].

## 3. Results and Discussion

**Figure 1:**
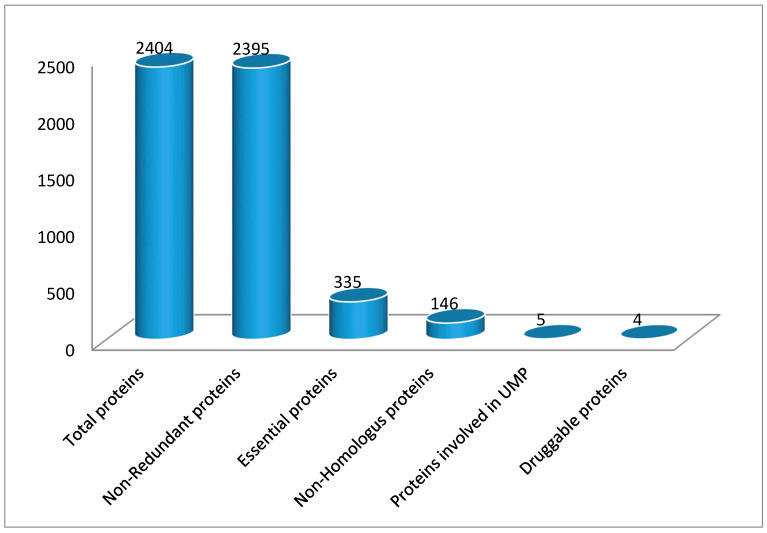
Summary for the detection of novel drug targets in *Staphylococcus saprophyticus*.

The major concern of our study was to identify the novel targets for designing potential drugs against *S. saprophyticus* strain ATCC 15305. Subtractive proteomic analysis of the entire *S. saprophyticus* proteome was performed by using various databases and computational tools. A brief overview of the subtractive proteomic procedure for finding the novel targets against the *S. saprophyticus* is illustrated in Figure 1.

### 3.1. Identification of Essential Proteins

The whole proteome of *S. saprophyticus* strain (ATCC 15305) was found to contain 2404 proteins. Out of these, 2395 non-redundant sequences were retrieved by using Cluster Database at High Identity with Tolerance (CD-HIT) at a 60% threshold. Non-redundant proteins in *S. saprophyticus* are in large numbers that are not necessary for the organism to survive and cannot be targeted directly. Essential proteins are the most promising targets for drug designing because the majority of antibacterial compounds are synthesized to harbor essential proteins. A total of 335 essential proteins were distinguished by using the Geptop 2.0 server.

### 3.2. Essential Non-Homologous Protein Identification

Proteins have an important role in various common cellular processes of bacteria and *H. sapiens* arose as homologs over the years. The proteins need to be critical for the pathogen’s survival within the body of the host but also non-homologous to host proteins for consideration as an effective drug target, and this condition is a requirement to avoid drug cross binding with the host proteins and the likelihood of the drug adverse events [26,27]. Therefore, BlastP was performed for all 335 essential proteins against *H. sapiens*. The results exhibited 146 non-homologous protein sequences having an identity of less than 30%. The best strategy for the generation of new drugs might be to develop and target inhibitors against these non-homologous sequences.

### 3.3. Metabolic Pathway Analysis

**Figure 2:**
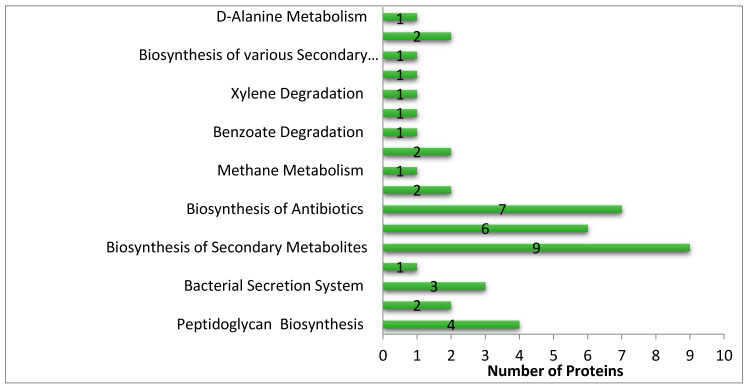
Distribution of different proteins in unique metabolic pathways of *S. saprophyticus.*

The analysis of the metabolic pathway of these 146 non-homologous protein sequences revealed that these proteins were involved in 54 pathways. To track drug targets involved in pathogen-specific pathways, a comparative study of the *S. saprophyticus* and *H. sapiens* metabolic pathways was carried out. The comparison of both the *S. saprophyticus* pathway and *H. sapiens* pathway exhibited that 17 pathways were pathogen-specific whereas the remaining 37 were found to be common in both pathogen and host. A total of 22 essential non-homologous proteins of *S. saprophyticus* were found involved in these 17 pathways. The distribution of proteins in each UMP is shown in Figure 2. We have classified these seventeen UMPs based on biochemical processes: metabolism pathways (70%), human disease pathways (12%), environmental information processing pathway (12%), cellular processes pathway (6%).

These 22 proteins were further analyzed by using the KEGG database (Table 1). Out of these 22 proteins, 5 were revealed to be associated entirely with unique pathways whereas the other 17 proteins were associated with multiple pathways. Moreover, these 17 proteins were also involved in certain pathways that were present in both the host as well as the pathogen, therefore these were excluded from further investigation. 

### 3.4. Subcellular Localization Prediction

Subcellular localization prediction provides a fast and relatively cost-effective method of obtaining information regarding the function of a particular protein. Moreover, it was found that proteins can localize at multiple sites, therefore localization is a critical aspect of designing any therapeutic agent [28]. Cytoplasmic proteins are considered more suitable as drug targets because proteins located on the membrane are difficult to purify and analyze [26]. The results of PSORTb and BUSCA revealed that all five proteins fall in the category of cytoplasmic proteins (Table 2).

### 3.5. Druggability of Therapeutic Targets 

Another important requirement for therapeutic targets is druggability. It is defined as the probability that a small-molecule drug modulates the activity of a therapeutic target protein [29,30]. The druggability of the non-homologous proteins of *S. saprophyticus* was detected by comparing their sequence similarities with drug targets, using the database of DrugBank. This resulted in the detection of four *S. saprophyticus* proteins that represented high similarity to FDA-approved small-molecule drugs.

### 3.6. Pathways Specific to S. saprophyticus in Comparison with H. sapiens

Four proteins, i.e., UDP-N-acetylenolpyruvoylglucosamine reductase, UDP-N-acetylmuramoylalanine-D-glutamate ligase, D-alanine-D-alanine ligase (DdIA), and alanine racemase, were found as the potent druggable targets. These four proteins were involved in three UMPs. Proteins present in the UMPs can also be considered pathogen specific and can serve as potential target for drugs and vaccines [26,31].

A major challenge to be dealt with during the selection of potential targets is to find out whether the targeted metabolic pathway is specific to bacteria. The three pathways that are unique to bacteria include peptidoglycan biosynthesis, vancomycin resistance, and D-alanine metabolism.

### 3.7. Peptidoglycan Biosynthesis 

Peptidoglycan makes up the bacterial cell wall, and the inhibitors that inhibit peptidoglycan are grouped in one of the major antimicrobial classes. The drug targets exhibiting the potential of inhibiting the biosynthesis of peptidoglycan can also minimize the pathogenicity caused by a microbe [32]. Three proteins, i.e., D-alanine ligase, D-glutamate ligase, and UDP-N-acetylenolpyruvoylglucosamine reductase, were indicated to be potential targets against the pathogen.

### 3.8. Vancomycin Resistance 

Vancomycin, a glycopeptide antimicrobial agent, is effective against the majority of gram-positive bacteria. It has the potential of inhibiting the peptidoglycan synthesis in cell walls of bacteria by forming an interaction with D-alanyl-D-alanine moieties and prevents their binding to the peptidoglycan chain [33]. 

### 3.9. D-Alanine Metabolism

The DdIA of this pathway has not shown any similarity with any host protein. D-ala is an important precursor of peptidoglycan biosynthesis pathway in bacterial cells. This enzyme is abundant in prokaryotes and is not present in eukaryotes (with some exceptions), which makes it a probable target for antibiotics development. Designing the inhibitors against this pivotal enzyme in peptidoglycan synthesis can result in the loss of structural integrity of bacterial cell walls as well as osmotic lysis [34].

The druggable proteins identified in our study are novel drug targets in *S. saprophyticus* and can be employed to design drugs. These drug targets need to be experimentally validated. Molecular modeling and virtual screening for these targets can be valuable in the development of potential therapeutic agents against *S. saprophyticus* and can help combat multidrug resistance. 

## 4. Conclusions

As resistance against all the available antimicrobials has been reported in the majority of gram-positive bacteria, there is an urgent need of developing new agents against novel drug targets. The present study has found four potent druggable proteins as novel targets in *S. saprophyticus* that can be utilized for developing drugs against them as these all play a role in pathogen-specific metabolic pathways and have also been targeted effectively in other microorganisms. The novel drug targets found may have moved on to the early stages of the drug design phase for the possible screening of new therapeutic candidates and are therefore proposed as an antimicrobial therapy for *S. saprophyticus.*

## Figures and Tables

**Table 1:** Essential non-homologous proteins involved in 17 unique metabolic pathways (UMPs).

Protein Name (Protein ID)	Common Pathway	Unique Pathway
**UDP-N-acetylmuramoyl-L-alanyl-D-glutamate-L-lysine ligase (Q49WE7)**		ssp00550-Peptidoglycan biosynthesis
**Penicillin-binding protein 1 (Q49WW3)**	ssp01100-Metabolic pathways	ssp00550-Peptidoglycan biosynthesis ssp01501-beta-Lactam resistance
**Protein translocase subunit SecE (Q49V45)**	ssp03060-Protein export	ssp03070-Bacterial secretion system
**Aspartokinase (Q49XJ5)**	ssp01210-2-Oxocarboxylic acid metabolism ssp01230-Biosynthesis of amino acids ssp00260-Glycine, serine, and threonine metabolism ssp00270-Cysteine and methionine metabolisms ssp00300-Lysine biosynthesis ssp01100-Metabolic pathways	ssp0026-Lobactam biosynthesis ssp01110-Biosynthesis of secondary metabolites ssp01120-Microbial metabolism in diverse environments ssp01130-Biosynthesis of antibiotics
**Penicillin-binding protein 2 (Q49XQ6)**	ssp01100-Metabolic pathways	ssp00550-Peptidoglycan biosynthesis ssp01501-beta-Lactam resistance
**Homoserine dehydrogenase (Q49XB5)**	ssp01230-Biosynthesis of amino acids ssp00260-Glycine, serine, and threonine metabolism ssp00270-Cysteine and methionine metabolisms ssp00300-Lysine biosynthesis ssp01100-Metabolic pathways	ssp01110-Biosynthesis of secondary metabolites ssp01120-Microbial metabolism in diverse environments ssp01130-Biosynthesis of antibiotics
**Protein translocase subunit SecY (Q49ZE8)**	ssp03060-Protein export	ssp03070-Bacterial secretion system
**Fructose-bisphosphate aldolase (Q49Z72)**	ssp01200-Carbon metabolism ssp01100-Metabolic pathways ssp01230-Biosynthesis of amino acids ssp00051-Fructose and mannose metabolism ssp00010-Glycolysis/Gluconeogenesis ssp00030-Pentose phosphate pathway	ssp01120-Microbial metabolism in diverse environments ssp01110-Biosynthesis of secondary metabolites ssp01130-Biosynthesis of antibiotics ssp00680-Methane metabolism
**Riboflavin synthase alpha chain (Q49YJ8)**	ssp00740-Riboflavin metabolism ssp01100-Metabolic pathways	ssp01110-Biosynthesis of secondary metabolites
**Glutamine synthetase (Q49XA2)**	ssp01100-Metabolic pathways ssp01230-Biosynthesis of amino acids ssp00220-Arginine biosynthesis ssp00250-Alanine, aspartate, and glutamate metabolism ssp00630-Glyoxylate and dicarboxylate metabolism ssp00910-Nitrogen metabolism	ssp01120-Microbial metabolism in diverse environments ssp02020-Two-component system
**Riboflavin biosynthesis protein RibD (Q49YJ9)**	ssp00740-Riboflavin metabolism ssp01100-Metabolic pathways	ssp01120-Microbial metabolism in diverse environments ssp02024-Quorum sensing
**Beta sliding clamp (Q4A179)**	ssp00220-Arginine biosynthesis ssp00310-Lysine degradation ssp00360-Phenylalanine metabolism ssp00630-Glyoxylate and dicarboxylate metabolism ssp00010-Glycolysis/Gluconeogenesis ssp00020-Citrate cycle (TCA cycle) ssp00040-Pentose and glucuronate interconversions ssp00053-Ascorbate and aldarate metabolism ssp00061-Fatty acid biosynthesis ssp00071-Fatty acid degradation ssp00250-Alanine, aspartate, and glutamate metabolism ssp00260-Glycine, serine, and threonine metabolism ssp00280-Valine, leucine, and isoleucine degradation ssp00330-Lysine biosynthesis ssp00350-Arginine and proline metabolism ssp00361-Tyrosine metabolism ssp00562-D-Glutamine and D-glutamate metabolism ssp00620-Inositol phosphate metabolism ssp00622-Pyruvate metabolism ssp00650-Propanoate metabolism ssp00660-Butanoate metabolism ssp00997-Nicotinate and nicotinamide metabolism ssp01100-Metabolic pathways ssp01210-2-Oxocarboxylic acid metabolism ssp01212-Fatty acid metabolism ssp03030-DNA replication ssp03430-Mismatch repair ssp03440-Homologous recombination	ssp00362-Benzoate degradation ssp00300-Chlorocyclohexane and chlorobenzene degradation ssp00471-Xylene degradation ssp00640-C5-Branched dibasic acid metabolism ssp00760-Biosynthesis of various secondary metabolites-Part 3 ssp01110-Biosynthesis of secondary metabolites ssp01120-Microbial metabolism in diverse environments ssp01130-Biosynthesis of antibiotics
**Putative preprotein translocase subunit (Q49Y76)**	ssp03060-Protein Export	ssp02024-Quorum sensing ssp03070-Bacterial secretion system
**DAHP synthetase-chorismate mutase (Q49YG8)**	ssp01230-Biosynthesis of amino acids ssp01100-Metabolic pathways ssp00400-Phenylalanine, tyrosine, and tryptophan biosynthesis	ssp01110-Biosynthesis of secondary metabolites ssp01130-Biosynthesis of antibiotics
**Putative heptaprenyl diphosphate synthase component (Q49XS4)**	ssp00900-Terpenoid backbone biosynthesis	ssp01110-Biosynthesis of secondary metabolites
**Malonyl CoA-acyl carrier protein transacylase (Q49X14)**	ssp00061-Fatty acid biosynthesis ssp01212-Fatty acid metabolism ssp01100-Metabolic pathways	ssp01110-Biosynthesis of secondary metabolites ssp01130-Biosynthesis of antibiotics
**Enoyl-[acyl-carrier-protein] reductase [NADPH](Q49WE0)**	ssp00061-Fatty acid biosynthesis ssp01212-Fatty acid metabolism ssp01100-Metabolic pathways	ssp01110-Biosynthesis of secondary metabolites ssp01130-Biosynthesis of antibiotics
**Chromosomal replication initiator protein DnaA (Q4A180)**		ssp02020-Two-component system
**UDP-N-acetylenolpyruvoylglucosamine reductase (Q49VT7)**		ssp00550-Peptidoglycan biosynthesis
**D-alanine-D-alanine ligase (Q49Z31)**		ssp01502-Vancomycin resistance ssp00473-D-Alanine metabolism ssp00550-Peptidoglycan biosynthesis
**UDP-N-acetylmuramoylalanine-D-glutamate ligase (Q49WW5)**		ssp00550-Peptidoglycan biosynthesis
**Alanine racemase (Q49Z24)**		ssp01502-Vancomycin resistance

**Table 2:** Subcellular localization prediction of proteins involved in UMPs.

Protein ID	Subcellular Localization	Whether Druggable
Q4A180	Cytoplasmic	No
Q49VT7	Cytoplasmic	Yes
Q49Z31	Cytoplasmic	Yes
Q49WW5	Cytoplasmic	Yes
Q49Z24	Cytoplasmic	Yes
